# Development and Initial Validation of a Frontline Health Worker mHealth Assessment Platform (MEDSINC^®^) for Children 2–60 Months of Age

**DOI:** 10.4269/ajtmh.18-0869

**Published:** 2019-04-15

**Authors:** Barry A. Finette, Megan McLaughlin, Samuel V. Scarpino, John Canning, Michelle Grunauer, Enrique Teran, Marisol Bahamonde, Edy Quizhpe, Rashed Shah, Eric Swedberg, Kazi Asadur Rahman, Hosneara Khondker, Ituki Chakma, Denis Muhoza, Awa Seck, Assiatta Kabore, Salvator Nibitanga, Barry Heath

**Affiliations:** 1 University of Vermont Robert Larner College of Medicine, Vermont Children’s Hospital, Burlington, Vermont;; 2 THINKMD, Inc., Burlington, Vermont;; 3 Northeastern University, Boston, Massachusetts;; 4 Physicians Computer Company, Winooski, Vermont;; 5 Universidad San Francisco de Quito, Quito, Ecuador;; 6 University of San Francisco de Quito- Ecuador Ministry of Health-Affiliate, Quito, Ecuador;; 7 Save the Children – US, Fairfield, Connecticut;; 8 Save the Children – International Bangladesh, Dhaka, Bangladesh;; 9 UNICEF-Burkina Faso, Ouagadougou, Burkina Faso

## Abstract

Approximately 3 million children younger than 5 years living in low- and middle-income countries (LMICs) die each year from treatable clinical conditions such as pneumonia, dehydration secondary to diarrhea, and malaria. A majority of these deaths could be prevented with early clinical assessments and appropriate therapeutic intervention. In this study, we describe the development and initial validation testing of a mobile health (mHealth) platform, MEDSINC^®^, designed for frontline health workers (FLWs) to perform clinical risk assessments of children aged 2–60 months. MEDSINC is a web browser–based clinical severity assessment, triage, treatment, and follow-up recommendation platform developed with physician-based Bayesian pattern recognition logic. Initial validation, usability, and acceptability testing were performed on 861 children aged between 2 and 60 months by 49 FLWs in Burkina Faso, Ecuador, and Bangladesh. MEDSINC-based clinical assessments by FLWs were independently and blindly correlated with clinical assessments by 22 local health-care professionals (LHPs). Results demonstrate that clinical assessments by FLWs using MEDSINC had a specificity correlation between 84% and 99% to LHPs, except for two outlier assessments (63% and 75%) at one study site, in which local survey prevalence data indicated that MEDSINC outperformed LHPs. In addition, MEDSINC triage recommendation distributions were highly correlated with those of LHPs, whereas usability and feasibility responses from LHP/FLW were collectively positive for ease of use, learning, and job performance. These results indicate that the MEDSINC platform could significantly increase pediatric health-care capacity in LMICs by improving FLWs’ ability to accurately assess health status and triage of children, facilitating early life-saving therapeutic interventions.

## INTRODUCTION

In 2016, the World Health Organization (WHO) and United Nationals Children’s Fund (UNICEF) estimated that 99% of all deaths of children younger than 5 years, approximately 5.6 million, occurred in low- and middle-income countries (LMICs).^1^ More than 50%, ∼ 3.0 M, of these deaths include clinical conditions such as pneumonia (respiratory failure), dehydration secondary to diarrheal diseases, malaria, and sepsis. A majority of these deaths could be prevented with early clinical risk assessments and appropriate basic therapeutic intervention.^2^ Unfortunately, access to quality health care in LMICs is significantly restricted because of a growing shortage of health-care professionals per population and limitations on health-care delivery infrastructure. The WHO recommends a minimum density threshold of 44.5 skilled health professionals/10,000 people to provide basic health coverage.^3^ Presently, 83 countries fall below this threshold, and it is estimated that 40–50 million new health workers will need to be trained by 2030 to attain an effective range of primary care services to ensure quality health-care delivery.^3^

To address these health-care challenges and improve childhood mortality, ministries of health and international aid agencies have been expanding the implementation of frontline health workers (FLWs)–based task-sharing health-care delivery programs.^4^ Such programs educate and train FLWs to provide basic health-care services to the most vulnerable communities where the shortage of health-care professionals is highest and services are most limited.^4^ Increasingly, these task-sharing programs are integrating digital mobile health (mHealth) platforms as a solution to expand health-care capacity by increasing medical knowledge, clinical skill sets, and case management guidelines to FLW.^5,6^ A majority of mHealth platforms for children have digitalized, to a varying extent, the WHO Integrated Management of Childhood Illnesses (IMCI) and integrated community case management (iCCM) protocols developed by the WHO and UNICEF.^7^ Implementation of IMCI has been shown to improve the consistency and quality of health assessments of children younger than 5 years in health facilities, whereas the goal of iCCM implementation at the community level using community health worker cohorts is to increase health-care coverage and access to basic treatment (WHO/UNICEF Joint Statement on iCCM).^8^ Implementation of these digital protocols on smartphones or tablets by FLW has shown the potential to increase the number of children being checked for important clinical danger signs, improved antibiotic use, and referrals for consultations and hospitalizations.^5,9,10^ A clinician-based study in Tanzania demonstrated that digitalized modified IMCI-ALMANACH algorithms in conjunction with selective point-of-care tests achieved a reduction of 43% in the proportion of clinical failures, a 58% reduction in the proportion of severe adverse events, and a substantial decrease in the proportion of antibiotic prescriptions from 30% to 11% compared with the traditional IMCI-ALMANACH algorithms.^11^ Despite these important improvements, current IMCI-iCCM–based and other mHealth platforms have not demonstrated consistency with implementation or a high agreement with expert clinical assessments of children with important clinical conditions such as pneumonia (26–41%).^5,12^ In addition, because of continued challenges with respect to usability and acceptability, many mHealth platforms have low sustainable adoption, data acquisition accuracy, workflow, and guideline compliance that require high initial and maintenance training and monitoring to sustain effectiveness.^13^ Although evidence for the importance and effectiveness of implementing mHealth solutions is increasing, their overall effect on impact is still being determined.^14,15^

Here, we describe the development of a next-generation FLW mHealth point-of-care clinical assessment, triage, treatment, and recommendation platform, MEDSINC, that incorporates WHO-based IMIC-iCCM protocols and guidelines as well as additional evidenced-based medicine into a “physician-centered clinical assessment approach” using Bayesian pattern recognition logic. This approach is unique in that it is not based on binary decision tree protocol methodology in which data and logic assignment are limited to “yes versus no” decisions, that can lead to misdiagnoses by inappropriate interpretation and emphasis on single clinical data points to determine a clinical assessment and severity. MEDSINC logic is based on the clinical assessment approaches by health-care professionals that include acquisition of a constellation of clinical data points about past and current history, symptoms, vital signs, and physical examination findings that are then “weighted” for their clinical significance and severity compared with normal values, followed by a cluster-pattern data analysis as they pertain to specific clinical conditions (e.g., dehydration) and disease risk (e.g., dysentery) to generate an integrated clinical risk assessment.

To gain insights into MEDSINC’s clinical logic accuracy, as well as usability and acceptability, we performed field-based testing in three countries, Burkina Faso, Ecuador and Bangladesh, by comparing MEDSINC-generated clinical assessments by FLWs with independent blinded “gold standard” clinical assessments by local health-care professionals (LHPs) assessing the same children.

## MATERIALS AND METHODS

### Algorithm and platform development.

**Figure 1.**
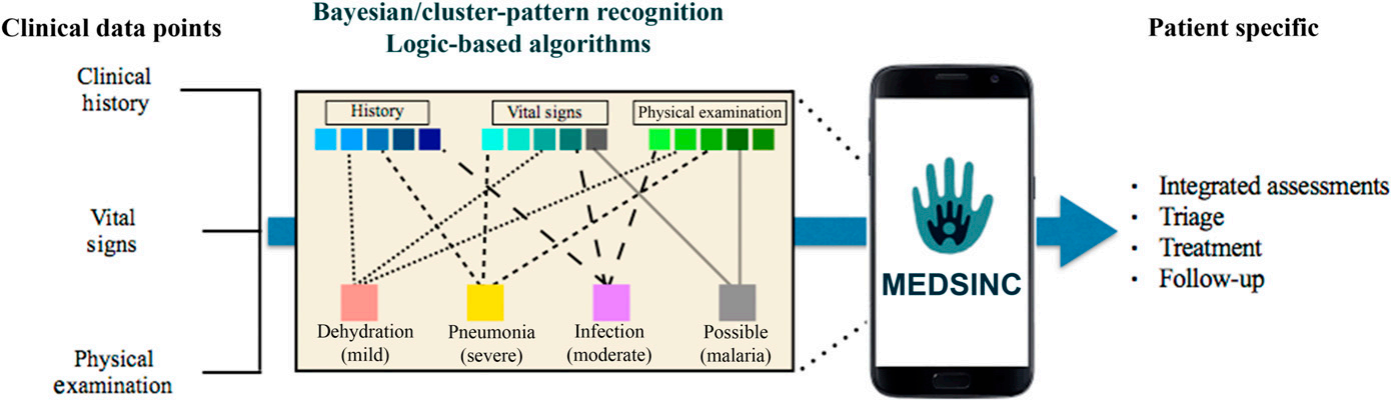
MEDSINC Bayesian/cluster-pattern algorithms use acquired clinical data points (see Table 1) that are given a numerical weighted score and then grouped based on clinical assessment patterns being processed. Severity assessments (none–moderate–severe) are then generated by unique tolerance scores for respiratory distress, dehydration, sepsis risk, and acute malnutrition. Clinical risk for eight additional clinical conditions—malaria, urinary tract infection, measles, anemia, cellulitis, ear infection, meningitis, and dysentery—are based on individual-based scores. MEDSINC platform also generates patient-specific triage, treatment, and follow-up recommendations. This figure appears in color at www.ajtmh.org.

The MEDSINC platform acquires and digitalizes key evidence-based data points that are then analyzed through physician-based logic to generate integrated clinical risk assessments, triage, treatment, and follow-up recommendations. The platform interprets 42 key clinical data points based on the WHO IMCI-iCCM guidelines and protocols, as well as other evidence-based data points that allow for expansion of the clinical conditions/diseases evaluated by MEDSINC. The platform guides users through a complete assessment, obligating the user to sequentially answer all questions using supportive embedded demonstration and training animated gif illustrations to enhance and improve the quality of data point acquisition. A summary of the acquired data points is summarized in Table 1. MEDSINC’s clinical logic is based on Bayesian weighting of each data point followed by cluster-pattern analysis for each specific disease or clinical conditions (Figure 1). Each data point is provided a numerical “weighted” score based on the degree of variance from normal database values or degree of clinical severity and then assigned into one or more of the eight disease assessment groups (malaria, measles, skin infections, meningitis, otitis media, dysentery, urinary tract infection, and anemia) in which risk versus no risk is determined based on assigned weighted data tolerance scores for each specific disease. In addition, aggregated scores of these data points are further analyzed to determine clinical severity risk (none/mild, moderate, and severe) for key clinical conditions, respiratory distress/pneumonia, dehydration, sepsis–systemic inflammatory response syndrome (SIRS) risk, and acute malnutrition that are based on sliding tolerance severity score thresholds. Therefore, the initial iteration of this platform generates 20 integrated clinical assessments. Based on the generated clinical assessments and severity risk, MEDSINC also creates the WHO IMC-iCCM compliant triage, treatment, and follow-up recommendations that are specific for age and weight.

**Table 1:** Clinical data points used by MEDSINC Bayesian/cluster-pattern recognition algorithms

Demographics	History of illness	Vital signs	Physical examination
Age	Concern that the child is very sick	Weight	Level of consciousness
Gender	Fever	Temperature	Skin turgor
	Seizure	Heart rate	Capillary refill
	Bloody stools	Respiratory rate	Infant suck
	Ear pain or discharge	Oxygen saturation (if available)	Nasal flaring or retractions
	Pain with urination		Chest indrawing
	Foul-smelling urine		Head bobbing
	Body ache/pain		Wheezing
	Headache		Pitting edema feet
	Difficulty breathing		Pale eyelids
	Cough		Pale palms
	Vomiting frequency (past 24 hours)		Pain moving neck
	Diarrhea frequency (past 24 hours)		Skin red, warm, swelling, pain, and discharge
	Drinking or breastfeeding		Nasal discharge
	Tears when crying		Conjunctivitis
	Last urination		Rash
	Sleeping pattern		MUAC

MUAC = mid-upper arm circumference.

Of importance is that the MEDSINC platform is engineered to be fully functional with or without access to cellular/wireless connectivity and is operating system agnostic, which allows it to be used on any mobile device with a touch screen. MEDSINC is also highly configurable for clinical content and regional localization (e.g., language, clinical diseases, treatment, user interface (UI)/user experience (UX), and treatment protocols) with the ability to quickly provide updates to reflect changing program/national guidelines for local customization.

### Validation testing.

#### Study test sites.

Validation test sites were determined by regional collaborating testing groups in concert with the Ministry of Health (MOH). This included two remote regional village sites in Burkina Faso (Yako and Gourcy districts); four regional sites (urban, costal, highland, and amazon) in Ecuador (Quito, Perdernales, Sigchos, and Coca); and the Rayer Bazar urban slum region in Dhaka, Bangladesh. Each testing site received appropriate approval by their ethics committees. THINKMD was granted approval through the University of Vermont’s Institutional Review Board for research in Burlington, VT; UNICEF-Burkina Faso received approval and authorization from the local MOH, Directorate of Maternal and Child Health in Burkina Faso; Save the Children-Bangladesh received approval and authorization from the organization’s internal ethics committee based at Save the Children headquarters in Washington, DC; and Universidad San Francisco de Quito (USFQ)/MOH-Ecuador received their approval from USFQ, Comité de Ética de Investigación en Seres Humanos in Quito.

#### MEDSINC training.

Field-based validation studies were performed following a standardized “train-the-trainer” approach with slight site-specific modifications based on collaborator request, the available technology at testing locations, number of attendees, and previous training of FLWs. Initial MEDSINC training occurred with LHPs and included a presentation on the background, functionality, and full use of the MEDSINC platform, in addition to working through standardized test cases, including metronome rate training to simulate heart and respiratory rate acquisition, and live cases using colleagues as patients. This approach using identical content was then repeated by the LHP for FLW training in groups of two to four trainees. Average time for each training group (ranging from 10 to 20 individuals) was between 4 and 6 hours, depending on the number of individuals.

#### MEDSINC validation.

**Figure 2.**
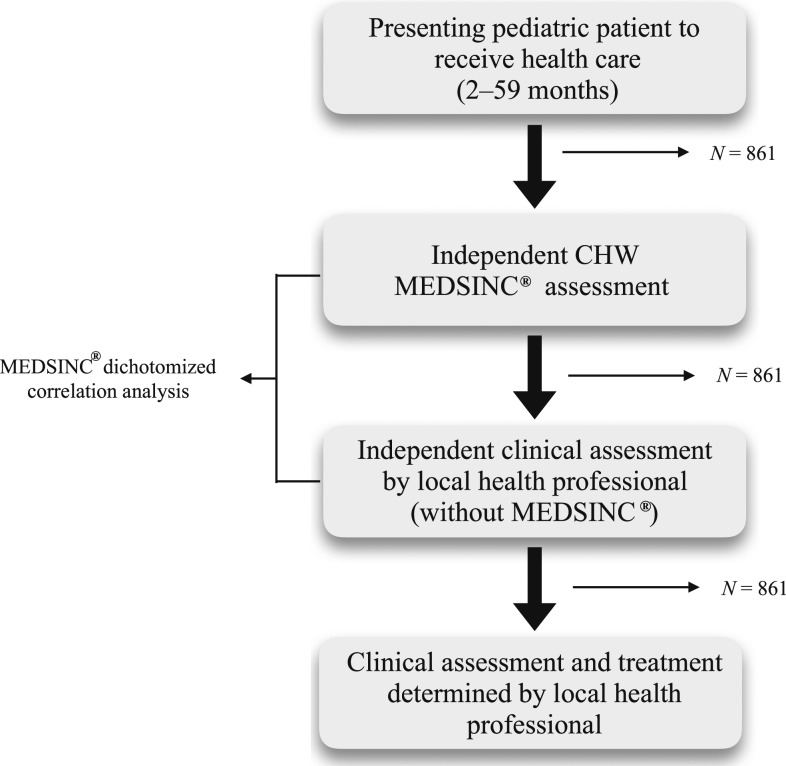
Validation study design and recruitment of subjects.

A focused validation study design was used with local testing partners and is outlined in Figure 2. All subjects, 2–60 months of age, were enrolled during FLW home visits as part of community outreach or at presentation to local/regional clinic for routine or acute care health-care visits. Parental consent was required and granted verbally at all study sites. MEDSINC assessments were performed on Apple iPod touch (Apple Inc., Cupertino, CA) (Burkina Faso and Ecuador) or Lenovo Yoga 8 Tablets (Lenovo Group Ltd., Beijing, China) (Bangladesh). Based on the MEDSINC version used at each site, severity risk correlations (respiratory distress, dehydration, sepsis–SIRS, and acute malnutrition) were evaluated at all three sites, whereas additional new specific disease risk assessments (malaria, dysentery, meningitis, ear infection, skin infection, anemia, measles, and urinary tract infection) developed during these validation studies were also evaluated during Ecuador and Bangladesh validation testing.

All subjects initially received an independent MEDSINC (offline) clinical assessment by FLWs over a 4- to 21-day study period depending on the size of the cohort and timeframe determined by in-country testing partners. Following each FLW MEDSINC clinical assessment, each subject was then independently evaluated and assessed by LHPs, who were blinded to the MEDSINC assessments generated by FLWs, using their personal standardized clinical approaches. The care and triage recommendations for each subject enrolled in validation studies were determined by LHPs based on their clinical assessments. All assessments were stored locally on the devices until internet connections were established. Data were transferred daily to the Health Insurance Portability and Accountability Act (HIPPA)-approved secure data management THINKMD server.

#### Data storage and security.

Data were protected and stored on International Business Machines Cloudant Database as a Service. All access to Cloudant databases is encrypted via HTTPS and passed through a RESTfull application programming interface with full authentication. There were nightly backups and replication of the databases.

#### Usability testing.

Assessment of MEDSINC UI and UX was performed using questionnaire surveys completed by participants through interviews conducted by local testing staff following completion of each local validation study. Feedback was acquired from all participating stakeholders, including FLWs, LHPs, MOH staff, local and regional program staff, and child caregivers, throughout all study periods and sites.

#### Statistical methods.

Dichotomized correlation analysis, as well as sensitivity, specificity, positive predictive value (PPV), and negative predictive value (NPV), was performed between independent FLW-generated MEDSINC assessments compared with independent LHP physician “gold standards,” generated clinical assessments of the same child. We estimated the mean and 95% credible intervals using Bayesian inference, with a binomial likelihood and a Jeffrey’s prior, that is, a beta distribution with both parameters equal to 0.5.

Inter-rater reliability analysis between MEDSINC-generated assessments by FLWs and LHPs were performed using both Cohen’s kappa statistics,^16,17^ and Gwet’s agreement coefficient (AC_1_)^18–20^ analysis because our data include unbalanced classes. Gwet’s AC_1_ is considered a more reliable indicator of diagnostic reliability than Cohen’s kappa because it relaxes the assumption made by Cohen’s kappa that each evaluator is independent, and as a result, Gwet’s AC_1_ does not suffer from the paradox of Cohen’s kappa, where there is high agreement, but a low kappa statistic.^18–20^ Support for the Gwet’s AC_1_ reliability analysis was confirmed by using stochastic simulations.

#### mHealth evidence reporting and assessment.

mHealth evidence reporting and assessment guidelines were adhered to for developing, testing, and reporting.^21^

## RESULTS

A total of 861 individual assessments of children aged 2–60 months were completed by 49 FLWs and 22 LHPs whose demographic composition is summarized in Tables 2 and 3. There was significant variability in the average years’ experience as a FLW (1–10) and education level. The LHP composition was 87% physicians (junior to senior) and 13% specially trained MOH pediatric health practitioners. The time to perform MEDSINC clinical assessments by FLWs during testing after performing 10–15 assessments was 5–10 minutes per child.

**Table 2:** Demographic summary of FLWs and LHPs participating in validation studies

Country	Number	Age (average years)	Experience (average years)	Education level
FLWs		27–38	1–10	
Bangladesh*	4	27	7	100% higher education and secondary school
Burkina Faso†	23	38	5–10	9% no formal education, 62% primary, and 29% secondary
Ecuador‡	22	28	1–5	82% higher education
LHPs				
Bangladesh	2	–	–	Physician
Burkina Faso	5	–	–	Physician (3) and health agent§ (2)
Ecuador	15	–	–	Physician

FLW = frontline health worker; LHP = local health-care professional; NGO = non-governmental organization; TAP = Técnicos en Atención Primaria en Salud.

* FLWs were represented by NGO-trained community organizers (4).

† FLWs were represented by Ministry of Health–trained community health workers (23); 21 completed surveys.

‡ FLWs were represented by community health workers called TAPs (15), medical students (2), and nurses (5).

§ Health agents, “Agent de Sante,” represent specialized health workers who provide primary health services and consultations for children and adults.

**Table 3:** Field-testing sites

Country	Location	Site description
Bangladesh	Dhaka	Urban slum
Burkina Faso	Yako	Rural/remote clinics and community homes
	Gourcy	Rural/remote clinics and community homes
Ecuador	Quito	Urban clinic
	Pedernales	Temporary/mobile health clinics in costal earthquake-effected region
	Sigchos	Rural clinics and homes in the highland region of the Andes
	Joyas de los Sachas/Coca	Rural hospitals in the Amazon Basin

### Clinical assessment correlation between MEDSINC and LHP.

**Figure 3.**
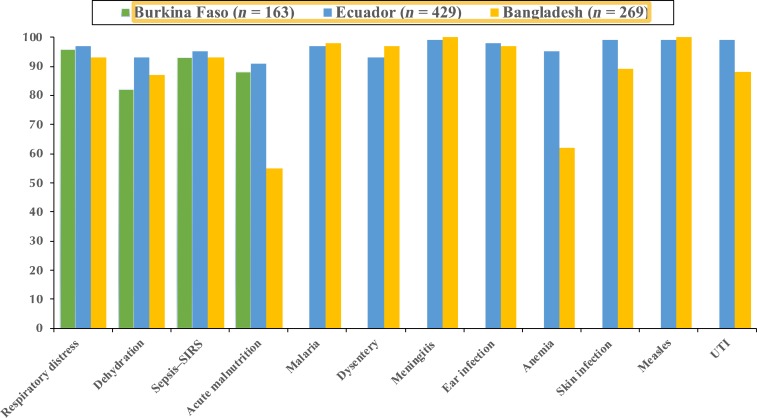
The overall correlation of MEDSINC-generated clinical assessments by non–health-care professionals with an average of 2 hours of training compared with local health-care professionals performing independent blinded clinical assessments of the same patient. This figure appears in color at www.ajtmh.org.

The distribution of FLW clinical MEDSINC assessments was as follows: Burkina Faso, *n* = 163; Ecuador, *n* = 429; and Bangladesh, *n* = 269. A summary of the overall percent correlation between MEDSINC assessments compared with LHP-independent clinical assessments of the same child is shown in Figure 3 and Table 4. With respect to the four clinical severity assessments (pneumonia/respiratory distress; dehydration; sepsis–SIRS; and acute malnutrition), we observed between 82% and 97% correlation between FLW-generated MEDSINC assessments and those generated by LHP. There was one outlier in which there was 55% correlation between MEDSINC and LHP for acute malnutrition in Bangladesh test cases. For the additional eight disease risk assessments tested in Ecuador and Bangladesh, we observed between 88% and 100% correlation between MEDSINC and LHP. There was one assessment outlier in the Bangladesh test case group, in which we observed a 62% correlation between MEDSINC and LHP for anemia risk assessment.

### Statistical analysis between FLW-generated MEDSINC clinical assessments and LHPs.

A summary of the statistical analyses is shown in Table 4. We observed between 85% and 100% comparative specificity for the four severity risk assessments and between 89% and 100% specificity for the eight disease risk assessments in the Ecuador and Bangladesh studies. As expected, there were two outliers as described for the clinical correlations, a 75% specificity for acute malnutrition and a 63% specificity for anemia risk during our Bangladesh studies. The ranges for sensitivity for both the clinical severity and disease risk assessments were quite varied, from 0.03 for dysentery (Ecuador) and cellulitis (Bangladesh) to 0.86 for respiratory distress/pneumonia severity (Bangladesh). This wide variability in sensitivity is expected because of the low prevalence of these clinical conditions in the test population because subjects were not selected for acute illnesses or clinical state at the time they sought health care.

Positive predictive value and NPV analyses correlated with the specificity, sensitive observations, with NPV ranges for the four severity risk assessments being between 0.94 and 1, with the one exception being 0.49 for acute malnutrition (Bangladesh). For the eight disease risk assessments, the observed NPV range was between 0.89 and 1 (Table 4). The range for PPV for all assessments was quite variable, between 0.09 and 1, correlating with the low prevalence in the population tested. We observed similar results with sensitivity and specificity for all our dichotomized comparisons (Table 4).

Inter-rater reliability analysis revealed clinical assessments agreement between MEDSINC and LHP greater than would be expected because of chance, with Gwet’s AC_1_ coefficients ranging between 0.79 and 0.97 (mean: 0.89). This metric ranges from 0 to 1, with a value of one indicating perfect agreement. Cohen’s kappa analysis comparisons varied and ranged from −0.008 to 0.48.

### Triage recommendation correlation between MEDSINC and LHP.

**Figure 4.**
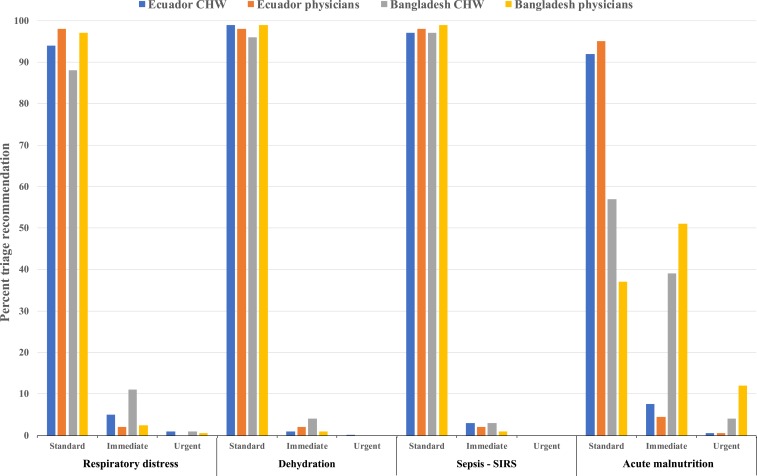
A comparison of the percent distribution of “standard–immediate–urgent” triage recommendations for respiratory distress, dehydration, sepsis–systemic inflammatory response syndrome, and acute malnutrition by the MEDSINC platform generated by FLWs compared with local health professionals for Ecuador and Bangladesh field studies. This figure appears in color at www.ajtmh.org.

A key component of frontline clinical assessments and subsequent medical intervention is the appropriate triage determination with respect to level and urgency. The MEDSINC platform provides three levels of triage recommendations (standard, immediate, and urgent care) which correspond with the IMCI-iCCM triage recommendations (green, yellow, and red), based on the platform-generated frontline clinical risk assessments by FLWs. The percent distribution of the MEDSINC triage recommendations for the four severity risk assessments for children assessed by FLWs compared with LHP triage recommendations of the same subjects during our Ecuador and Bangladesh testing is shown in Figure 4. These data were not available from our testing in Burkina Faso because they were not included in the platform during our validation studies at this site. We observed very high correlations for percent distribution correlations for all three MEDSINC-generated triage recommendations by FLWs and those by LHPs, in particular for children requiring standard care versus immediate or urgent care. MEDSINC platform triage recommendations were in general more conservative for children identified with the risk of respiratory distress-pneumonia and sepsis–SIRS, whereas LHP recommended more children for both immediate and urgent care for acute malnutrition in our Bangladesh field studies than those at our Ecuador test sites. The differences in the triage recommendations for acute malnutrition in the Bangladesh study also reflects the difference seen with this assessment as described earlier.

### MEDSINC usability and acceptability.

**Figure 5.**
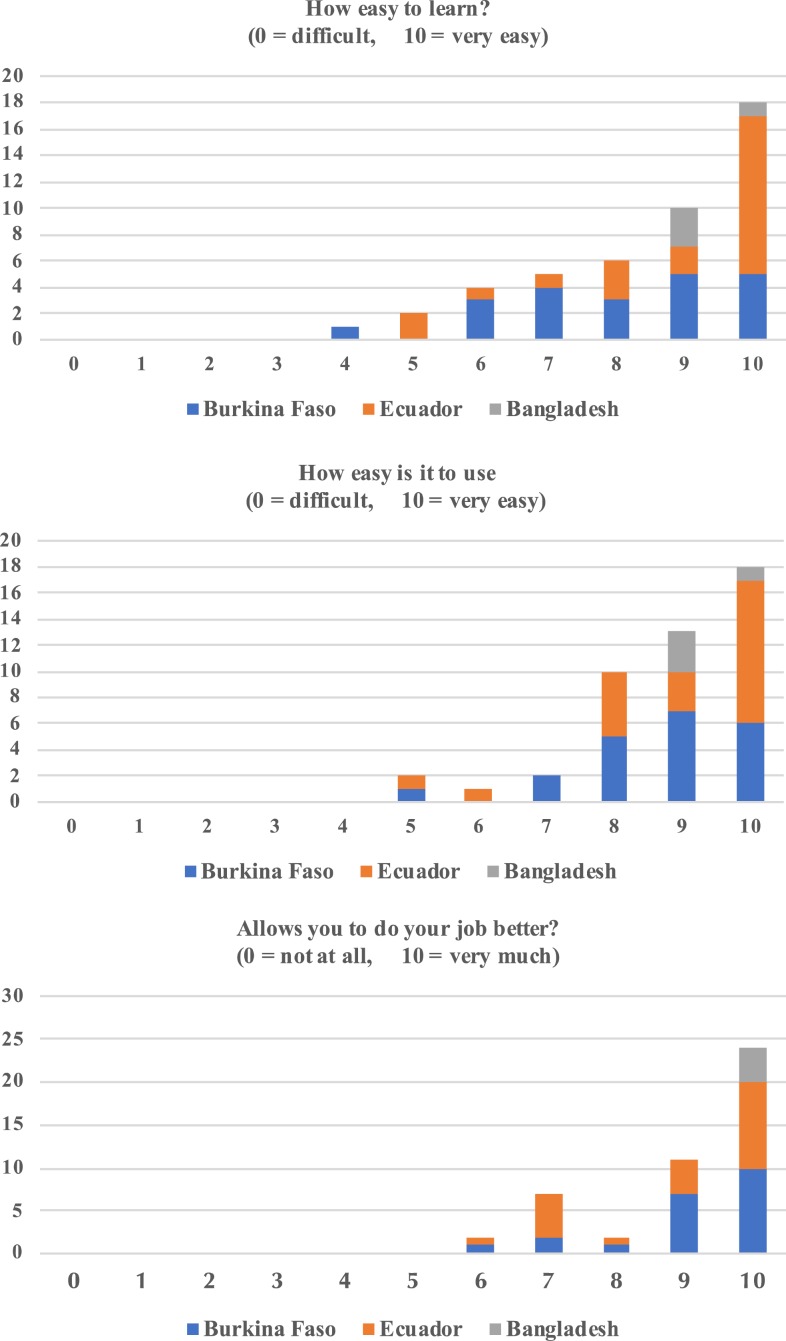
The distribution of responses to usability and acceptability surveys by frontline health workers. This figure appears in color at www.ajtmh.org.

Demographic classification for 47/49 FLWs (96%) and 22 LHPs (100%) exhibited significant diversity in age, experience, education, and previous use of digital mHealth technology (Table 2). Usability and acceptability responses from FLWs with respect to how easy the MEDSINC platform was to learn, use, and perform a job better are summarized in Figure 5. Frontline health worker also noted that they believe MEDSINC would lead to better health care for children, improve their skills, enable them to identify a sick child, help them to conduct a complete assessment, increase their adherence to IMCI guidelines, and make them feel more professional and respected (data not shown). No concerns were made during these validation studies by FLW regarding the overall time required to complete each clinical assessment and how it may affect their workflow. They did share that with time, using the MEDSINC platform became easier and that they felt more confident and performed more complete assessments.

Parent/caregivers noted that MEDSINC would enable them to receive health care in the community, which would significantly help with health-care access and cost for time lost for work and transportation.

Importantly, LHPs noted that MEDSINC would ameliorate major challenges they face, including early detection and treatment of children with critical diseases in their communities and not at overburdened local and regional health clinics, in particular allowing physicians to see those children who are the sickest. Participating health professionals and staff from MOHs noted that MEDSINC would help detect problems early for pediatric patients, providing the opportunity to potentially better manage outbreaks and provide timelier initial treatment.

## DISCUSSION

Quality point-of-care mHealth technology would provide a key tool for expanding health-care access, especially in LMICs, by assisting FLWs with identifying patients who are sick, leading to appropriate triage and facilitating early life-saving therapeutic interventions. This would ultimately lead to a decrease in premature deaths and disabilities (disability-adjusted life years). Two key components required for successful adoption and scaling of mHealth systems are the ability to scale while maintaining the quality of clinical assessments and usability/acceptability by end users. The data presented here indicate that the MEDSINC clinical assessment logic, triage recommendations, and UI meet the essential standards required for mHealth technology adoption and scaling.

Our validation studies demonstrate high overall clinical correlations and specificity between MEDSINC evaluations by FLWs compared with independent LHP “gold standard” assessments with the exceptions of two outliers, anemia and acute malnutrition in the Rayer Bazar slum studies in Bangladesh. Of interest, local epidemiology survey prevalence studies for acute malnutrition and anemia in this slum highly correlated with the MEDSINC assessment prevalence values as opposed to the LHP prevalence values (Table 5). This suggests the MEDSINC assessments by FLWs outperformed those by LHPs. This may be the consequence of the MEDSINC platforms UI directing FLWs to acquire additional clinical data and perform standardized physician-based examinations for each encounter that check for conjunctival pallor for anemia and MUAC or weight acquisition for acute malnutrition.

**Table 4:** Comparative statistical analysis between FLHW-generated MEDSINC clinical assessments to independent clinical assessments by LHPs

	Correlation (%)*	Specificity (95% CI)	Sensitivity (95% CI)	PPV (95% CI)	NPV (95% CI)	GAC† (95% CI)	Kappa
Burkina Faso (*n* = 163)							
Clinical severity assessment							
Respiratory distress	96	0.84 (0.78–0.89)	0.44 (0.13–0.75)	0.12 (0.02–0.23)	0.98 (0.95–0.99)	0.74 (0.60–0.87)	0.19 (0.04–0.33)
Dehydration	82	0.99 (0.99–1)	0.21 (0.001–0.39)	0.5 (0.06–0.94)	0.97 (0.95–0.99)	0.90 (0.83–0.97)	0 (NA)
Sepsis–SIRS	93	0.98 (0.95–0.99)	0.30 (0.09–0.52)	0.5 (0.12–0.80)	0.94 (0.90–0.97)	0.93 (0.88–0.99)	0.52 (0.32–0.72)
Acute malnutrition	88	0.93 (0.88–0.97)	0.70 (0.51–0.88)	0.57 (0.39–0.75)	0.96 (0.93–0.99)	0.80 (0.69–0.91)	0.28 (0.08–0.48)
Disease risk assessment							
Malaria	–	–	–	–	–	–	–
Meningitis	–	–	–	–	–	–	–
Anemia	–	–	–	–	–	–	–
Urinary tract infection	–	–	–	–	–	–	–
Dysentery	–	–	–	–	–	–	–
Ear infection	–	–	–	–	–	–	–
Measles	–	–	–	–	–	–	–
Cellulitis (skin infection)	–	–	–	–	–	–	–
Ecuador (*n* = 429)							
Clinical severity assessment							
Respiratory distress	97	0.96 (0.94–0.97)	0.72 (0.45–0.97)	0.27 (0.11–0.45)	0.98 (0.96–0.99)	0.97 (0.95–0.99)	0.37 (0.29–0.45)
Dehydration	93	0.99 (0.98–0.99)	0.23 (0.02–0.46)	0.42 (0.08–0.77)	0.98 (0.96–0.99)	0.97 (0.95–0.98)	0.25 (0.16–0.35)
Sepsis–SIRS	95	0.97 (0.95–0.99)	0.31 (0.04–0.61)	0.18 (0.02–0.37)	0.99 (0.97–0.99)	0.96 (0.94–0.98)	0.21 (0.11–0.30)
Acute malnutrition	91	0.93 (0.91–0.96)	0.45 (0.25–0.66)	0.27 (0.13–0.42)	0.97 (0.95–0.98)	0.90 (0.86–0.93)	0.29 (0.20–0.38)
Disease risk assessment							
Malaria	97	0.97 (0.96–0.99)	0.17 (0.00–0.57)	0.04 (0.00–0.17)	0.99 (0.98–0.99)	0.97 (0.95–0.99)	0 (0–0.06)
Meningitis	99	0.99 (0.99–1)	0.25 (0.00–0.77)	0.25 (0.00–0.77)	0.99 (0.99–1)	0.99 (0.99–1.0)	0 (0–0.09)
Anemia	95	0.97 (0.95–0.99)	0.34 (0.13–0.57)	0.32 (0.12–0.54)	0.97 (0.96–0.99)	0.94 (0.92–0.97)	0.3 (0.20–0.39)
Urinary tract infection	99	0.98 (0.96–0.99)	0.69 (0.39–0.96)	0.37 (0.14–0.60)	0.99 (0.98–0.99)	0.97 (0.95–0.99)	0.46 (0.37–0.56)
Dysentery	93	0.99 (0.99–1)	0.05 (0.00–0.13)	0.75 (0.22–1)	0.93 (0.91–0.96)	0.93 (0.90–0.96)	0.06 (0.03–0.10)
Ear infection	98	0.99 (0.97–0.99)	0.5 (0.17–0.83)	0.39 (0.10–0.68)	0.99 (0.98–0.99)	0.98 (0.96–0.99)	0.42 (0.32–0.51)
Measles	99	0.99 (0.99–1)	0.25 (0.00–0.77)	NA (0.00–1)	0.99 (0.99–1)	0.99 (0.99–1)	0 (NA)
Cellulitis (skin infection)	99	0.99 (0.99–1)	0.07 (0.00–0.26)	NA (0.00–1)	0.98 (0.97–0.99)	0.98 (0.97–0.99)	0 (NA)
Bangladesh (*n* = 269)							
Clinical severity assessment							
Respiratory distress	93	0.90 (0.86–0.93)	0.81 (0.56–0.99)	0.20 (0.07–0.34)	0.99 (0.98–1.00)	0.88 (0.84–0.93)	0.28 (0.19–0.38)
Dehydration	87	0.97 (0.94–0.99)	0.37 (0.01–0.77)	0.15 (0.00–0.36	0.99 (0.98–0.99)	0.96 (0.93–0.98)	0.15 (0.05–0.26)
Sepsis–SIRS	93	0.96 (0.94–0.98)	0.12 (0.00–0.44)	0.05 (0.00–0.19)	0.98 (0.97–0.99)	0.95 (0.92–0.98)	0 (0–0.09)
Acute malnutrition	55	0.75 (0.66–0.83)	0.54 (0.46–0.61)	0.78 (0.70–0.86)	0.49 (0.41–0.57)	0.24 (0.12–0.36)	0.26 (0.15–0.37)
Disease risk assessment							
Malaria	98	0.98 (0.96–0.99)	NA (0.00–1.00)	0.08 (0.00–0.30)	0.99 (0.99–1.00	0.98 (0.96–0.99)	0 (NA)
Meningitis	100	0.99 (0.99–1.00)	NA (0.00–1.00)	NA (0.00–1.00)	0.99 (0.99–1.00)	NaN	NaN
Anemia	62	0.63 (0.57–0.69)	0.10 (0.00–0.36)	0.005 (0.00– 0.02)	0.99 (0.95–0.99)	0.45 (0.34–0.57)	0 (0–0.01)
Urinary tract infection	88	0.88 (0.84–0.92)	0.70 (0.35–0.99)	0.10 (0.02–0.20)	0.99 (0.98–1.00)	0.86 (0.80–0.91)	0.14 (0.06–0.21)
Dysentery	97	0.99 (0.98–1)	0.12 (0.00–0.44)	0.25 (0.00–0.77)	0.99 (0.97–0.99)	0.98 (0.97–0.99)	0 (0–0.1)
Ear infection	97	0.98 (0.96–0.99)	0.5 (0.17–0.83)	0.39 (0.10–0.68)	0.99 (0.97–0.99)	0.97 (0.94–0.99)	0.41 (0.29–0.53)
Measles	100	0.99 (0.99–1.00)	NA (0.00–1.00)	NA (0.00–1.00)	0.99 (0.99–1.00)	NaN	NaN
Cellulitis (skin infection)	89	0.99 (0.99–1.00)	0.05 (0.00–0.13)	0.75 (0.23–0.1.00	0.89 (0.85–0.93)	0.88 (0.83–0.92)	0.06 (0.02–0.10)

CI = credible interval; GAC = Gwet’s AC_1_; LHP = local health-care professional; NPV = negative predictive value; PPV = positive predictive value; SIRS = systemic inflammatory response syndrome; FLHW = frontline health worker; CI = confidence interval; NA = not available; NaN = not a number.

* Percent correlation between MEDSINC-generated clinical severity and risk assessments by frontline health workers compared with independent clincal assessments by LHPs.

† GAC Inter-rater reliability (CE 95% [low–high]).

**Table 5:** Comparison of FLWs (MEDSINC), LHPs, and survey-generated prevalence data from Bangladesh field site

	Bangladesh		
	Prevalence (MEDSINC)	Prevalence (LHPs)	Prevalence (survey)
Clinical severity assessments			
Respiratory distress	0.12	0.03	0.08*
Dehydration	0.04	0.01	NA
Sepsis–SIRS	0.04	0.01	NA
Acute malnutrition	**0.43**	**0.63**	0.43*
Clinical risk assessments			
Malaria	0.02	0.002	0.002*
Meningitis	0.002	0.002	NA
Anemia	**0.36**	**0.02**	**0.22**†
Urinary tract infection	0.13	0.02	0.002*
Dysentery	0.006	0.01	NA
Ear infection	0.03	0.02	0.04*
Measles	0.002	0.002	0.002*
Cellulitis (skin infection)	0.006	0.11	0.02‡

FLW = frontline health worker; LHP = local health-care professional; SIRS = systemic inflammatory response syndrome; icddr,b = International Centre for Diarrhoeal Disease Research, Bangladesh; SEARO = South East Asia Regional Office.

* Urban Health Survey.

† National Micronutrient Status Survey 2011–12; icddr,b, UNICEF, Bangladesh, Global Alliance for Improved Nutrition and the Institute of Public Health and Nutrition.

‡ General prevalence of health-care–seeking behavior of slum dwellers in Dhaka city. Results of a household survey, WHO/SEARO/Country Office for Bangladesh and Health Economics Unit, Ministry of Health and Family Welfare, Bangladesh, 2015.

There is presently a limited number of FLW-generated IMCI clinical assessment comparison validation studies for children aged 2 months to 5 years that used physician-based assessments as a gold standard. Horwood et al.^12^ compared IMCI-based clinical assessments by trained health workers with those by IMCI experts and showed correlations ranging between 27.8% and 66.7% (pneumonia-40.6%, severe pneumonia-47.8%, no dehydration-66.7%, some dehydration-37.8%, severe dehydration-33.3%, suspected meningitis-36.4%, and severe malnutrition-27.8%). Other studies have shown overall IMCI versus physician correlations to be 36.7%,^22^ 58%,^23^ and 45%.^24^ Specific IMCI-based clinical disease correlations versus physician assessments that were observed were 76% respiratory, 67% dehydration, 50% fever, 31% anemia, and 38% acute malnutrition.^24^ Rambaud-Althaus et al.^5^ demonstrated the overall health worker clinical assessments for serious conditions using the ALMANCH platform validated by clinicians also using ALMANCH to range between 33% and 86% (malaria-86%, pneumonia-33%, dysentery-75%, and urinary tract infection-67%).

Despite the limitations in comparing these types of studies and their inherent differences, it appears that the MEDSINC platform had significantly higher, more consistent clinical correlations to LHP physician-based “gold standard.” Using a study design that uses LHP clinical assessments as the “gold standard” for dichotomized correlations has limitations. Studies have shown that diagnostic errors and incorrect evaluations by physicians and health practitioners can range from 12% to 35%.^25,26^ In addition, multiple studies have observed intra- and interobserver clinical assessment correlations between physicians and specialty trained health professionals to range between 22% and 73%.^27–29^ Such variability between health-care professionals also likely contributed to our inter-rater reliability kappa value analysis (Table 4), thus supporting the Gwet’s AC_1_ approach as a more reliable inter-rater reliability analysis for this study design. A similar study conducted for personality disorder comparison with unbalanced classes, Gwet’s AC_1_ inter-rater reliability coefficients, was shown to be a more reliable indicator of diagnostic reliability than Cohen’s kappa.^19,20^ To further validate that Gwet’s AC_1_ is a more appropriate inter-rater reliability statistic for this study than Cohen’s kappa, we simulated patient encounters, where conditions were evaluated in silico by both a physician and MEDSINC (data not shown). However, for each run of the simulation, we fixed the level of agreement between the physician and MEDSINC. By varying the level of agreement—coupled with the empirical estimates of condition prevalence and diagnostic accuracy obtained at our three test sites—we evaluated whether the true, at least in the simulation, agreement was better summarized by Cohen’s kappa or Gwett’s AC_1_. As demonstrated in many previous studies, Gwett’s AC_1_ provided a substantially more accurate representation of the true diagnostic agreement. Gwet’s AC_1_ relaxes the assumption made by Cohen’s kappa that each evaluator is independent; as a result, Gwet’s AC_1_ does not suffer from the paradox of Cohen’s kappa, where there is high agreement, but a low kappa statistic.^18^

The initial validation approach we used for the MEDSINC platform can provide unique insights and potential value of this platform but has limitations as well. Additional validation, outcomes, and impact approaches will be critical to fully assess this, as well as other mHealth platforms being developed. Additional MEDSINC studies are ongoing and presently planned for locations where the prevalence of children with moderate–severe conditions is significantly higher to more accurately evaluate specificity, sensitivity, PPV, and NPV of MEDSINC assessments than LHPs.

In addition to the quality of clinical assessments, it is critical that digital health platforms have high usability and acceptability interfaces to achieve full adoption and scaling. Current IMCI-iCCM guideline-based digital protocols have been shown to require subjective decisions by FLW, as well as difficult to learn, maintain skills, and sustain knowledge base.^13^ As a result, implementation programs presently have to commit significant funds for training and supervision. Despite this support, achieving high rates of adherence to IMCI guidelines continues to be challenging. In a recent study, only 52.9% of children with cough, 18% with indrawing chest and tachypnea, and 73% with diarrhea received correct IMCI classifications at the time of referral.^30^ Improved coverage of antenatal services and immunizations by FLW has been demonstrated; unfortunately, such improvements have been difficult to sustain because of technical issues, lack of effective monitoring and supervision, social barriers, poor service/connection, low perceived usefulness of the intervention, and feeling of an increased workload.^31,32^ Such adoption issues have led to difficulties with mHealth scaling, impact, cost-effectiveness, efficacy, and feasibility.^33,34^

To address these challenges, the MEDSINC platform was developed to guide FLW users to acquire more than 85% of IMCI clinical data points for each encounter, in addition to other important evidence-based data points with respect to illness severity, mental status/lethargy, and cellulitis/skin infection; vital sign acquisition including heart rate; and oxygen saturation if available. Following data acquisition, the MEDSINC logic performs integrated assessments that include 87% of all IMCI individual assessments for each encounter while adding evaluation for cellulitis requiring antibiotic therapy. Thus, for each assessment, FLWs using the MEDSINC platform will acquire 87% of the requested IMCI data points and 87% of the IMCI guideline decision requests while receiving high marks for acceptability and usability. Preliminary evidence during an initial MEDSINC implementations in the Razer Bazar slum in Dhaka, Bangladesh, and in the Kano region of Nigeria supports these observations. Specifically, we have observed a sustainable 2-fold increase in the number of encounters per month, 2- to 8-fold increase in counseling sessions, and > 40% increase in danger sign acquisition following MEDSINC implementation (data not shown). Usability and acceptability data from FLWs and program directors have also been consistent or exceed those observed during the validation studies described here.

Despite these initial positive results, it is critical that long-term studies be developed to investigate whether the MEDSINC platform and other similar mHealth tools have the ability to promote and accelerate scaling of FLW-based primary health-care service delivery programs while maintaining quality and increased health-care capacity that will ultimately contribute to a decrease in premature deaths and disabilities in children living in LMICs.
